# Supplementary material for: Climate-assisted persistence of tropical fish vagrants in temperate marine ecosystems
Source: Commun Biol. 2021 Oct 28;4:1231. doi: 10.1038/s42003-021-02733-7 (PMC8553944; doi:10.1038/s42003-021-02733-7)
Supplement: Supplementary file 2 — Supplementary Information [file 42003_2021_2733_MOESM2_ESM.pdf]

Climate-assisted persistence of tropical fish vagrants in temperate marine ecosystems

Laura Gajdzik*, Thomas M. DeCarlo, Adam L. Koziol, Mahsa Mousavi-Derazmahalleh, Megan Coghlan, Matthew W. Power, Michael Bunce, David V. Fairclough, Michael J. Travers, Glenn I. Moore, and Joseph D. DiBattista.

*Corresponding author: laura.gajdzik@gmail.com

Table of contents

Supplementary Figure S1: Spatial autocorrelation between geographic and genetic distances
Supplementary Figure S2: Heatmap of read abundance for stomach contents of rabbitfish individuals
Supplementary Figure S3: Population stratification based on neutral SNP loci with fastSTRUCTURE
Supplementary Figure S4: Population stratification based on outlier SNP loci with fastSTRUCTURE
Supplementary Figure S5: Genetic groupings based on a discriminant analysis of principal components using neutral SNP loci
Supplementary Table S1: Pairwise genetic differentiation (F_{st})
Supplementary Table S2: Pairwise genetic differentiation (G_{st})
Supplementary Table S3: Relative genetic migration rates
Supplementary Table S4: Measures of genetic diversity

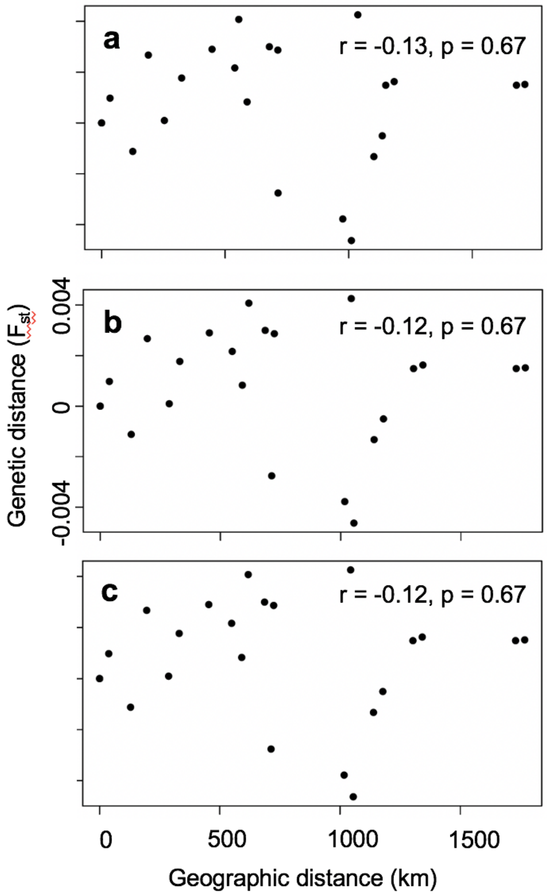

Supplementary Figure S1: Results of spatial autocorrelation analysis that shows no relationship between geographic (kilometers, km) and genetic (fixation index, F_{st}) distances for all sampled rabbitfish (*Siganus fuscescens*) from Kimberley to Cockburn Sound, which are tropical and temperate sites, respectively, separated by more than 1,500 km. Distance computed at a depth of **(a)** 0 m, **(b)** 5 m, and **(c)** 10 m.

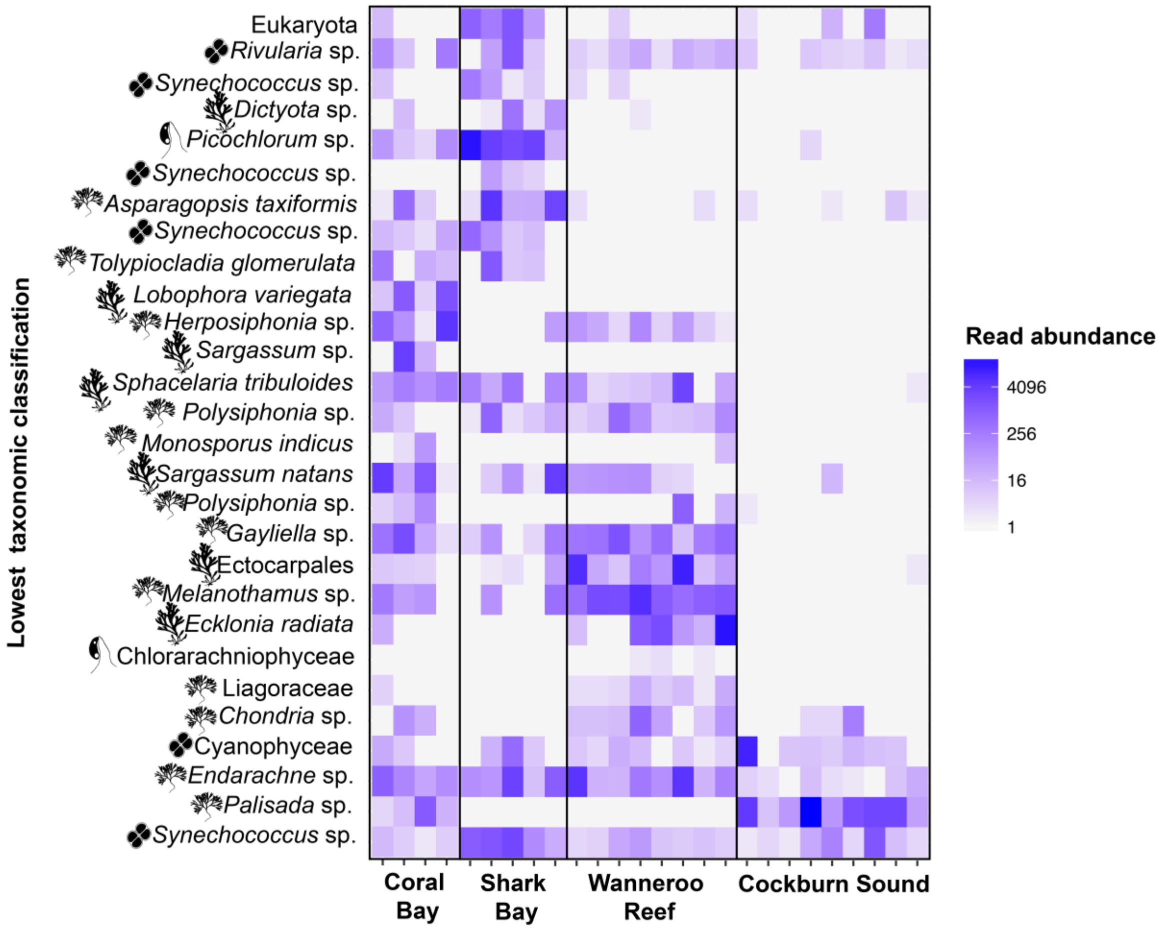

Supplementary Figure S2: Heatmap depicting the top 30% most abundant sequences across all stomach samples of rabbitfish from tropical/subtropical (Coral Bay and Shark Bay) and temperate sites (Wanneroo Reef and Cockburn Sound). The meaning of the silhouettes is defined on Figure 4.

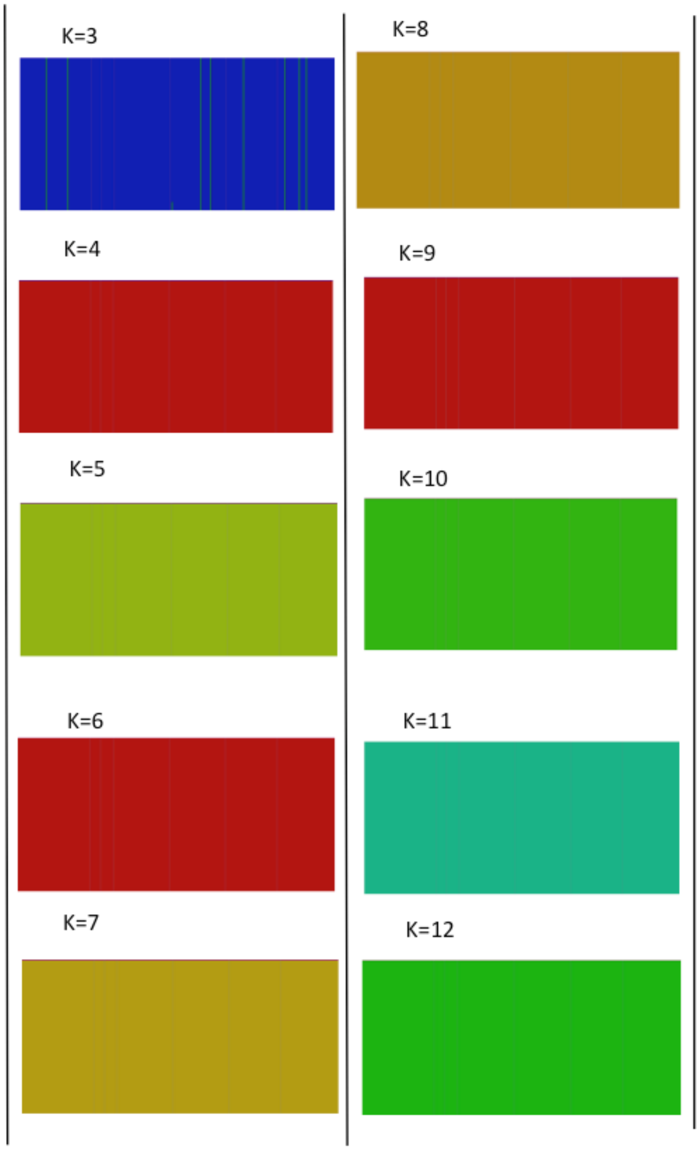

Supplementary Figure S3: Genetic structure among 220 *Siganus fuscescens* individuals sampled from seven regions along the west coast of Australia based on 5,507 neutral SNP loci using fastSTRUCTURE (*K*= 3 to *K* = 12). Each colour denotes a different population affiliation. Note that the colours are not equivalent among panels.

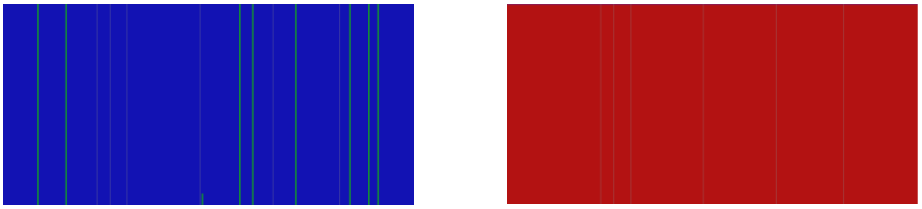

Supplementary Figure S4: Genetic structure (*K*= 3 on the left and *K*= 4 on the right) among 220 *Siganus fuscescens* individuals sampled from seven regions along the west coast of Australia based on 172 outlier SNP loci using fastSTRUCTURE. Each colour denotes a different population affiliation. Note that the colours are not equivalent among panels.

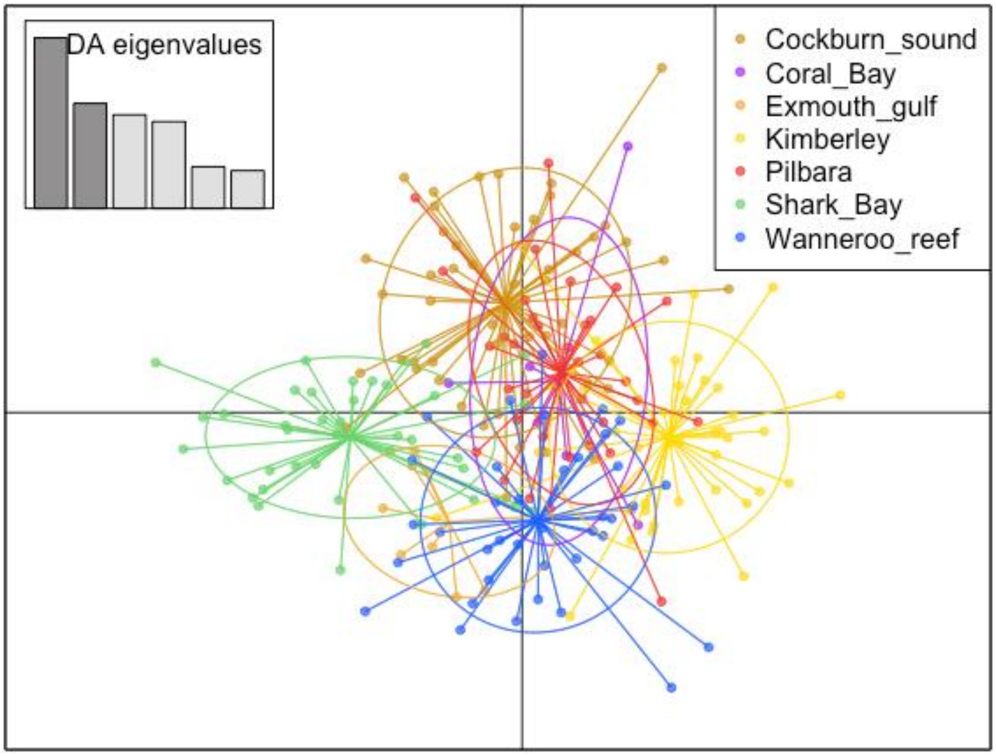

Supplementary Figure S5: Scatterplot of discriminant analysis of principal components (DAPC) performed on 220 *Siganus fuscescens* individuals sampled from seven regions along the west coast of Australia, with populations coloured by region. Dots represent individual genotypes and axes show the first two discriminant functions.

Supplementary Table S1: Pairwise genetic differentiation with F_{st} values (below the diagonal) and p-values (above the diagonal; in bold when significant after Narum correction) based on 5,507 neutral loci among 220 *Siganus fuscescens* rabbitfish individuals sampled from seven regions along the west coast of Australia.

Sample site	Kimberley	Pilbara	Exmouth Gulf	Coral Bay	Shark Bay	Wanneroo Reef	Cockburn Sound
**Kimberley**	-	**0.0008001**	0.3236324	0.9267927	**<0.0000001**	**0.0063006**	**0.0012001**
**Pilbara**	0.0021659	-	0.1034103	0.4921492	**<0.0000001**	0.0257026	0.0092009
**Exmouth Gulf**	0.0008289	0.0026730	-	0.6352635	0.0493049	0.7635764	0.6175618
**Coral Bay**	-0.0027650	0.0000959	-0.0011171	-	0.2213221	0.9719972	0.9825983
**Shark Bay**	0.0042578	0.0040774	0.0029015	0.0017668	-	**<0.0000001**	**<0.0000001**
**Wanneroo Reef**	0.0014875	0.0014861	-0.0013265	-0.0037780	0.0029985	-	0.0505051
**Cockburn Sound**	0.0015175	0.0016285	-0.000506	-0.0046316	0.0028675	0.0009777	-

Supplementary Table S2: Pairwise genetic differentiation (Nei's G_{st} values) based on 5,507 neutral SNP loci compared among 220 *Siganus fuscescens* rabbitfish individuals sampled from seven regions along the west coast of Australia. Significant genetic differentiation between population pairs (*i.e.*, when the lower 95% confidence interval [CI] did not overlap with zero) are indicated in bold.

**Pairwise population comparison**			**Actual G_{st} value**	**Lower CI interval**	**Upper CI interval**
Cockburn Sound	vs	Coral Bay	0.0019	-0.0116	0.0252
Cockburn Sound	vs	Exmouth Gulf	0.0027	-0.0081	0.0192
Cockburn Sound	vs	Kimberley	0.0016	-0.0003	0.0038
Cockburn Sound	vs	Pilbara	0.0019	-0.0002	0.0049
**Cockburn Sound**	**vs**	**Shark Bay**	**0.0022**	**0.0002**	**0.0046**
Cockburn Sound	vs	Wanneroo Reef	0.0015	-0.0006	0.0041
Coral Bay	vs	Exmouth Gulf	0.0056	-0.0146	0.0363
Coral Bay	vs	Kimberley	0.0027	-0.0109	0.0266
Coral Bay	vs	Pilbara	0.0048	-0.0094	0.0306
Coral Bay	vs	Shark Bay	0.0039	-0.0104	0.0289
Coral Bay	vs	Wanneroo Reef	0.0031	-0.0110	0.0283
Exmouth Gulf	vs	Kimberley	0.0033	-0.0078	0.02
Exmouth Gulf	vs	Pilbara	0.0047	-0.0069	0.0215
Exmouth Gulf	vs	Shark Bay	0.0035	-0.0073	0.0202
Exmouth Gulf	vs	Wanneroo Reef	0.0029	-0.0081	0.0197
Kimberley	vs	Pilbara	0.0022	-0.0003	0.0052
**Kimberley**	**vs**	**Shark Bay**	**0.0029**	**0.0006**	**0.0056**
Kimberley	vs	Wanneroo Reef	0.0018	-0.0004	0.0047
**Pilbara**	**vs**	**Shark Bay**	**0.0031**	**0.0005**	**0.0061**
Pilbara	vs	Wanneroo Reef	0.0021	-0.0005	0.0053
**Shark Bay**	**vs**	**Wanneroo Reef**	**0.0025**	**0.0002**	**0.0053**

Supplementary Table S3. Relative migration rates based on paiwise G_{ST} values computed with divMigrate from the R-package *diveRsity* using 10,000 permutations. Rabbitifish (*Siganus fuscescens*) source populations are shown in columns, sink populations are shown in rows. No significant asymmetric migration was found.

	**Kimberley**	**Pilbara**	**Exmouth Gulf**	**Coral Bay**	**Shark Bay**	**Cockburn Sound**	**Wanneroo Reef**
**Kimberley**	-	0.726	0.310	0.257	0.725	0.964	0.784
**Pilbara**	0.852	-	0.260	0.211	0.762	0.811	0.811
**Exmouth Gulf**	0.272	0.284	-	0.130	0.288	0.298	0.300
**Coral Bay**	0.214	0.219	0.123	-	0.234	0.242	0.236
**Shark Bay**	0.793	0.658	0.2687	0.222	-	0.856	0.752
**Cockburn Sound**	0.983	0.950	0.324	0.275	0.937	-	0.932
**Wanneroo Reef**	0.852	0.739	0.291	0.221	0.778	1.000	-

Supplementary Table S4. Number of individuals (N), mean allelic richness (A_r), mean heterozygosity (H_e), and mean observed heterozygosity (H_o) for 220 individuals of rabbitifish (*Siganus fuscescens*) sampled at seven sites along the coast of Western Australia. The tropical/subtropical sites are Kimberley, Pilbara, Exmouth Gulf, Coral Bay, and Shark Bay. The temperate sites (where vagrants were introduced) are Wanneroo Reef and Cockburn Sound.

Samples Sites	N	A_r	H_e	H_o
Kimberley	40	1.180	0.042	0.037
Pilbara	36	1.163	0.039	0.033
Exmouth Gulf	9	1.140	0.038	0.034
Coral Bay	7	1.123	0.035	0.032
Shark Bay	39	1.168	0.041	0.038
Wanneroo Reef	39	1.172	0.042	0.035
Cockburn Sound	50	1.181	0.043	0.037
