# Supplementary material for: Climate-assisted persistence of tropical fish vagrants in temperate marine ecosystems
Source: Commun Biol. 2021 Oct 28;4:1231. doi: 10.1038/s42003-021-02733-7 (PMC8553944; doi:10.1038/s42003-021-02733-7)
Supplement: Supplementary file 3 — Description of Supplementary Files [file 42003_2021_2733_MOESM3_ESM.pdf]

Description of Additional Supplementary Files

File name: Supplementary Data 1

Description: Presence/absence data and results of IndVal analysis representing all pairwise comparisons among all food items detected in the stomach contents of rabbitfish individuals from two sub/tropical sites (Coral Bay, CB, and Shark Bay, SB) and two temperate sites (Wanneroo Reef, WR, and Cockburn Sound, CS) in western Australia.

File name: Supplementary Data 2

Description: Presence and absence data as well as results of IndVal analysis (index, statistic and associated p-value and BH corrected p-values) representing significant pairwise comparisons among all food items found in the stomach contents of rabbitfish individuals sampled from two tropical sites (Coral Bay, CB, and Shark Bay, SB) and two temperate sites (Wanneroo Reef, WR, and Cockburn Sound, CS) in Western Australia.

File name: Supplementary Data 3

Description: Metadata for each Siganus fuscescens individual caught from 13 sites across seven regions in Western Australia (WA). Information for individual size (total length, TL and standard length, SL), gonad maturity stage (GMS) where the stages (Al-Marzouqi et al. 2011) are immature, maturing, mature, and ripe, sex (female, F and male, M), as well as which individuals were used in a published microbiome study by Jones et al. (2018) or in the present study for population genomics (pop gen) and dietary DNA metabarcoding.
