# Supplementary material for: Climate-assisted persistence of tropical fish vagrants in temperate marine ecosystems
Source: Commun Biol. 2021 Oct 28;4:1231. doi: 10.1038/s42003-021-02733-7 (PMC8553944; doi:10.1038/s42003-021-02733-7)
Supplement: Supplementary file 7 — Reporting Summary [file 42003_2021_2733_MOESM7_ESM.pdf]

nature research

Corresponding author(s): Laura Gajdzik
Last updated by author(s): 03/22/2021

Reporting Summary

Nature Research wishes to improve the reproducibility of the work that we publish. This form provides structure for consistency and transparency in reporting. For further information on Nature Research policies, see Authors & Referees and the Editorial Policy Checklist .

Statistics

For all statistical analyses, confirm that the following items are present in the figure legend, table legend, main text, or Methods section.

n/a	Confirmed	
☐	☒	The exact sample size (*n*) for each experimental group/condition, given as a discrete number and unit of measurement
☐	☒	A statement on whether measurements were taken from distinct samples or whether the same sample was measured repeatedly
☐	☒	The statistical test(s) used AND whether they are one- or two-sided<br>*Only common tests should be described solely by name; describe more complex techniques in the Methods section.*
☐	☒	A description of all covariates tested
☐	☒	A description of any assumptions or corrections, such as tests of normality and adjustment for multiple comparisons
☐	☒	A full description of the statistical parameters including central tendency (e.g. means) or other basic estimates (e.g. regression coefficient) AND variation (e.g. standard deviation) or associated estimates of uncertainty (e.g. confidence intervals)
☐	☒	For null hypothesis testing, the test statistic (e.g. *F*, *t*, *r*) with confidence intervals, effect sizes, degrees of freedom and *P* value noted<br>*Give P values as exact values whenever suitable.*
☐	☒	For Bayesian analysis, information on the choice of priors and Markov chain Monte Carlo settings
☒	☐	For hierarchical and complex designs, identification of the appropriate level for tests and full reporting of outcomes
☒	☐	Estimates of effect sizes (e.g. Cohen's *d*, Pearson's *r*), indicating how they were calculated

Our web collection on statistics for biologists contains articles on many of the points above.

Software and code

Policy information about availability of computer code

Data collection	DArTsoft14 software, Illumina Miseq platform, SODA v.3.3.
Data analysis	Geneious v.10.2.6, USEARCH v.10, VSEARCH, R v.3.5.3, BLASTN, fastSTRUCTURE v.1, MATLAB2012b

For manuscripts utilizing custom algorithms or software that are central to the research but not yet described in published literature, software must be made available to editors/reviewers. We strongly encourage code deposition in a community repository (e.g. GitHub). See the Nature Research guidelines for submitting code & software for further information.

Data

Policy information about availability of data

All manuscripts must include a data availability statement. This statement should provide the following information, where applicable:
- Accession codes, unique identifiers, or web links for publicly available datasets
- A list of figures that have associated raw data
- A description of any restrictions on data availability

MiSeq reads and DArTseq data are deposited in the Dryad repository: https://doi.org/10.5061/dryad.dr7sqv9xj Portion of the 23S rRNA gene of Ecklonia radiata was deposited on GenBank (accession number MW752516). Sea surface temperature data are available at https://www.esrl.noaa.gov/psd/data/gridded/data.noaa.oisst.v2.highres.html and https://coralreefwatch.noaa.gov/product/5km/.

Field-specific reporting

Please select the one below that is the best fit for your research. If you are not sure, read the appropriate sections before making your selection.

Ecological, evolutionary & environmental sciences study design

All studies must disclose on these points even when the disclosure is negative.

Study description	Two hundreds and two rabbitfish individuals (Siganus fuscescens) were collected from 13 sites and seven regions across western Australia (Supporting information Table S7) by researchers, the fisheries department of Western Australia, and recreational divers, with approved licenses, and with minimum handling and rapid euthanasia. Fin clips were taken and preserved in ethanol for the population genetic analysis. From thirty-three individuals, the stomach contents were also preserved in ethanol and use for the dietary DNA metabarcoding analysis. Ten IPCC models were used to project two temperature contours by 2100.
Research sample	A fish fin clip or a stomach content are each considered a research sample (Metadata available in the Supporting Information Table S7). The samples represent fish vagrants to temperate environments following the marine heatwave in 2010/2011 and tropical residents, and their population connectivity and feeding differences are intended to correspond to evolutionary and ecological responses of organisms facing rising global temperatures and acute climate events.
Sampling strategy	Sampling was conducted across 2/3 of the west coast of Australia in various habitats. The study was conducted to maximize the replication within budgetary, logistical, and permitting constraints. The sample size is deemed suitable compared to other DNA metabarcoding and genetic connectivity studies.
Data collection	Population genetic data using DArTseq (SNPs) was conducted by Andrzej Killian and data analyzes was done in R. Population stratification was performed using fastSTRUCTURE. Dietary DNA metabarcoding was performed at Curtin University (Australia). Read sequences were filtered using Geneious and Usearch, clustered in operational taxonomic units with Usearch and Vsearch, taxonomically assigned with BLASTN, and significant differences tested in R. Please see Method section for additional details.
Timing and spatial scale	Sampling was conducted between 2013 and 2017. The spatial extent of the study was over two thousands kilometers in Western Australia (Figure 1).
Data exclusions	No data were excluded.
Reproducibility	DNA extracts from fin clips and stomach contents are stored at Curtin University.
Randomization	No randomization was conducted.
Blinding	The ancestral population stratification analysis was ran without prior knowledge about geographical information as well as the taxonomic assignments of gut contents.

Did the study involve field work? ☒ Yes ☐ No

Field work, collection and transport

Field conditions	This study was conducted in Western Australia, characterized by a latitudinal gradient in environmental conditions from tropical to temperate environments. Appropriate measures for sampling were taken such as diving equipment for cold waters, calm conditions, and shark shield.
Location	This study was conducted in Western Australia across 16 degrees in latitude.
Access and import/export	DNA extractions of fish fin clips and stomach contents are stored at Curtin University (Western Australia). No import/export permits were required as the fish samples was conducted entirely within Australia.
Disturbance	Minimum touching or disturbances to other marine organisms was done when collecting fish.

Reporting for specific materials, systems and methods

We require information from authors about some types of materials, experimental systems and methods used in many studies. Here, indicate whether each material, system or method listed is relevant to your study. If you are not sure if a list item applies to your research, read the appropriate section before selecting a response.

Materials & experimental systems

n/a	Involved in the study	
☒	☐	Antibodies
☒	☐	Eukaryotic cell lines
☒	☐	Palaeontology
☐	☒	Animals and other organisms
☒	☐	Human research participants
☒	☐	Clinical data

Methods

n/a	Involved in the study	
☒	☐	ChIP-seq
☒	☐	Flow cytometry
☒	☐	MRI-based neuroimaging

Animals and other organisms

Policy information about studies involving animals; ARRIVE guidelines recommended for reporting animal research

Laboratory animals	Approved animal and ethics permits: #2632, #01-000039-1, and #AEC201527 (Please see additional info in the funding section)
Wild animals	Fish were sampled with minimum handling and pain, and humanely euthanized when brought back to the boat/shore.
Field-collected samples	Fish fin clips and gut contents were preserved in ethanol in the laboratory and their DNA extracted. These DNA extracts are stored in at Curtin University.
Ethics oversight	Approved by the Animal Ethic Committee : ethic permit #AEC201527

Note that full information on the approval of the study protocol must also be provided in the manuscript.
